# Arctiin suppresses H9N2 avian influenza virus-mediated inflammation via activation of Nrf2/HO-1 signaling

**DOI:** 10.1186/s12906-021-03462-4

**Published:** 2021-11-26

**Authors:** Beixian Zhou, Linxin Wang, Yueyun Liang, Jing Li, Xiping Pan

**Affiliations:** 1 Center of stem cell and Regenerative Medicine, The People’s Hospital of Gaozhou, Gaozhou, China; 2 Guangzhou Laboratory, No. 9, XingDaoHuanBei Road, Guangzhou International Bio Island, Guangzhou, 510005 China; 3 Department of Anesthesiology, The People’s Hospital of Gaozhou, Gaozhou, China; 4 State Key Laboratory of Respiratory Disease, National Clinical Research Center of Respiratory Disease, Guangzhou Institute of Respiratory Health, the First Affiliated Hospital of Guangzhou Medical University, Guangzhou Medical University, NO. 195, Dongfengxi Road, Guangzhou, 510120 China; 5 Institute of Chinese Integrative Medicine, Guangzhou Medical University, Guangzhou, Guangdong China

**Keywords:** *Arctium lappa L.*, lignan, arctiin, H9N2 avian influenza virus, anti-inflammatory

## Abstract

**Background:**

H9N2 avian influenza viruses (AIVs) infect avian and mammalian hosts and provide internal genes for new emerging highly pathogenic avian viruses that cause severe pneumonia with high mortality, for which few medications are available. Arctiin, a bioactive lignan glycoside, has been reported to possess multiple pharmacological properties. However, the effect of arctiin on H9N2 virus infection is unclear. In the current study, we analyzed the effect of arctiin on H9N2 virus infection and the underlying molecular mechanism in vitro.

**Methods:**

The antiviral effect against H9N2 virus was determined by plaque reduction assay (PRA) and progeny virus reduction assay. We employed MTT assay, qRT-PCR, ELISA, immunofluorescence and Western blotting to better understand the anti-inflammatory effect and corresponding mechanism of arctiin on H9N2 virus-infected cells.

**Results:**

The results showed that arctiin had antiviral activity against H9N2 virus. Arctiin treatment reduced H9N2 virus-triggered proinflammatory cytokines, such as IL-6, and TNF-α. Moreover, arctiin significantly suppressed H9N2 virus-mediated expression of COX-2 and PGE_2_. Furthermore, we found that arctiin inhibited H9N2 virus-mediated activation of RIG-I/JNK MAPK signaling. Interestingly, arctiin treatment obviously reversed H9N2 virus-induced reduction of Nrf2, increased the nuclear translocation of Nrf2, and upregulated Nrf2 signaling target genes (HO-1 and SOD2). Zinc protoporphyrin (Znpp)—an HO-1 inhibitor—weakened the inhibitory effect of arctiin on H9N2 virus-induced RIG-I/JNK MAPK and proinflammatory mediators.

**Conclusion:**

Taken together, these results suggested that the anti-inflammatory effects of arctiin on H9N2 virus infection may be due to the activation of Nrf2/HO-1 and blocked RIG-I/JNK MAPK signaling; thus, arctiin may be a promising agent for prevention and treatment of H9N2 virus infections.

**Supplementary Information:**

The online version contains supplementary material available at 10.1186/s12906-021-03462-4.

## Introduction

H9N2 avian influenza virus (AIV) was initially isolated in 1966 from turkeys in the United States [1]. It contains eight segments of single-stranded negative-sense RNA wrapped by a lipid bilayer envelope [1]. The natural hosts of H9N2 AIVs are wild birds and domestic poultry [2]. Although the infection with H9N2 virus in poultry usually causes subclinical to mild signs, it can also result in fatal outcomes in case of co-infection with bacteria or other microorganisms [3]. In recent years, the incidence of H9N2 virus cross-species transmission to humans has significantly increased. Serological surveys have revealed a high risk of H9N2 seropositivity in humans, indicating that H9N2 viruses have adapted to be able to infect humans [4]. It is remarkable that highly pathogenic avian influenza (e.g., H5N1 and H7N9) has acquired gene segments from H9N2 viruses, resulting in fatal respiratory diseases in humans [5, 6]. The pandemic potential of H9N2 virus raises serious public health risks. Novel therapeutic strategies for treatment of H9N2 virus should be developed continually.

Host cells express various intracellular pattern recognition receptors (PRRs) that are capable of recognizing viral components and driving activation of diverse host signaling pathways to dictate the host’s innate immune response against invading viruses [7]. Retinoic acid inducible gene-I (RIG-I), also known as DDX58, is a cytosolic PRR; it is well-characterized due to its crucial role in viral RNA sensing and initiating host defense [8]. Upon influenza virus (IV) infection, RIG-I engagement induces the activation of NF-κB and MAPKs (p38, ERK1/2, and JNK) signaling pathways, leading to the production of proinflammatory cytokines and chemokines, such as interferon beta (IFN-β), tumor necrosis factor alpha (TNF-α), interleukin 6 (IL-6), interleukin 8 (IL-8), and interferon-gamma induced protein 10 (IP-10) [9]. However, numerous reports have revealed that uncontrolled proinflammatory states driven by these continuous signaling cascades often result in detrimental effects on the human respiratory system or even death [10]. Concomitantly, to successfully establish replication, AIVs utilize these signaling pathways to aid in progeny viral entry, viral ribonucleoprotein complex (vRNP) nuclear export, translation, and virion budding [11–13]. Therefore, targeting the host signaling may be a promising therapeutic strategy for respiratory diseases elicited by influenza virus, including H9N2 virus.

The transcription factor, NF-E2-related factor 2 (Nrf2), plays a critical role in antioxidant and anti-inflammatory processes [14]. Upon various stimuli, Nrf2 dissociates from Kelch-like ECH-associated protein 1 (Keap1) and translocates into the nucleus, which is followed by the transcription of an array of anti-inflammatory and antioxidant genes, such as Heme oxygenase-1 (HO-1), superoxide dismutase 2 (SOD2), and NAD(P)H quinone oxidoreductase 1 (NQO1) [14]. Genetic or pharmacological activation of the Nrf2 pathway could exert multiple beneficial effects, including anti-inflammatory, neuroprotective, and antiviral effects [15–18]. According to previous studies, the anti-inflammatory effects of Nrf2 signaling have been linked to the suppression of NF-κB signaling [19]. Recent studies have demonstrated that the activation of Nrf2 provided protective effects against IV-mediated acute lung injury by suppressing viral replication and inflammation [15]. Increased expression of HO-1 has been found to limit IV replication via upregulation of interferon-induced antiviral effector proteins [20]. Therefore, we proposed that the activation of Nrf2 signaling by active compounds may help in the treatment of influenza-associated diseases.

*Arctium lappa L.* (burdock) is a medicinal plant with a number of pharmacological activities, including antihypertensive [21], antibacterial [22], antiviral [23], and anti-allergic effects [24]. Chemically, multiple bioactive compounds (e.g., lignan, flavonoid, polyphenol) have been extracted from *Arctium lappa L.* [25, 26]. Among these components, arctiin is a lignan glycoside that possesses various types of pharmacological properties [27]. For instance, arctiin has been shown to protect mice from lipopolysaccharide (LPS)-mediated acute lung injury via inactivation of PI3K/AKT signaling [28]. Arctiin was also found to increase virus-specific antibodies against influenza A (H1N1) virus [29]. However, the effects and the underlying mechanisms of arctiin against influenza virus infection have not yet been fully explored. In the current study, we aimed to investigate the effect of arctiin on H9N2 virus infection, but also the underlying molecular mechanism.

## Material and Methods

### Preparation of extracts

#### Generals

Silica gel 60 GF254 (Qingdao Haiyang) was used for vacuum liquid chromatography. Thin-layer Chromatography (TLC) was carried out using silica gel 60 F254 and RP-18 F254 plates (Merck). High-performance liquid chromatography (HPLC) was conducted using a Shimadzu system equipped with a LC-6 AD pump and a Diode Array Detector (SPD-M20A), as well as a Zorbax Eclipse XDB-C18 column (9.4 × 250 mm, 5 μm particle size, Agilent); mobile phase acetonitrile – water (9:1 v/v); flowrate 1 mL/min, injection volume 500 μL, wavelength 254 nm and 365 nm. HPLC solvents were purchased from Merck.

#### Extraction of arctiin

The plant materials were purchased from Bozhou traditional Chinese Medicine Market (Anhui provicine, China) and identified as dried seeds of *Arctium lappa L* by Professor Xiping Pan in Guangzhou laboratory. All solvents were purchased from Damao Chemical Reagent Factory (Tianjin, China). The dried seeds (2 kg) were extracted with 95% ethanol (3 × 4 L, 1 h each) in reflux. The ethanol solution was concentrated under reduced pressure to obtain a crude extract (165 g). The extract was chromatographed on a silica gel (200–300 mesh) column using dichloromethane - methanol mixed solvent as gradient eluent (from 10:1 to 1:1) to obtain fractions A–H. Fraction E was further purified by recrystallization to afford a white crystalline powder (58.6 mg), which was identified as the known compound arctiin by ^1^H and ^13^C NMR spectral data analysis.

### Cell culture

A549 cells and Madin–Darby canine kidney (MDCK) cells purchased from American Type Culture Collection (ATCC; Manassas, VA, USA) were stored in liquid nitrogen in our laboratory. After thawing in a 37 °C water bath, the cells were cultured at 37 °C in DMEM medium containing 10% fetal bovine serum.

### Virus and viral infection

AIVs A/Hongkong/Y280/97 (H9N2) were purchased from ATCC and propagated in the allantoic cavities of 10-day-old embryonated chicken eggs. The viral titers were determined in confluent monolayers of MDCK cells as described previously [30]. Briefly, cells were infected with 10-fold serial dilutions of the virus stocks, and then covered with a mixture of serum-free MDEM, 1% agarose and 1 μg/ml of Trypsine TPCK. After incubation at 37 °C for 48 h, cells were stained with 0.1% crystal violet for viral plaque count. Aliquots of H9N2 viruses were stored at −80 °C.

For viral infection, A549 cells were grown to confluent monolayers and then washed twice with PBS. Next, serum-free DMEM medium containing 0.1 multiplicities of infection (MOI) of H9N2 virus was added to the cells. After 2 h of incubation at 37 °C, the inoculum was replaced by fresh serum-free DMEM medium containing various concentrations of compounds for 24 h.

### Antiviral assays

To determine antiviral effect of arctiin against H9N2 virus, we used plaque reduction assay (PRA) and progeny virus reduction assay. For the PRA, monolayers of MDCK cells were inoculated with H9N2 virus (100 PFU/well) for 2 h, and then replaced with DMEM containing 0.8% agarose with or without indicated concentration of arctiin. After 48 h, the cells were fixed by 10% paraformaldehyde and stained with 0.1% crystal violet for viral plaques’ visualization. For progeny virus reduction assay, A549 cells were infected with H9N2 virus (MOI = 0.1) in the presence or absence of arctiin for 24 h. Then, progeny virus titers in the culture supernatants were measured on MDCK cells.

### Cell viability assay

The viability of A549 cells with indicated concentration of arctiin treatment was evaluated using 3-(4,5-dimethylthiazol-2-yl)-2,5-diphenyltetrazolium bromide (MTT) assay. In brief, A549 cells (1 × 10^5^ cells per well) were grown onto 96-well plates and cultured overnight at 37 °C in 5% CO_2_. The culture supernatants were discarded and replaced with mediums containing arctiin of various concentrations (0–800 μg/mL) for 24, 48 and 72 h. After that, 10 μL of MTT solution (5 mg/mL) was added to each well and incubated at 37 °C for additional 4 h. Then, the culture medium was discarded and 100 μL of DMSO was added to each well for formazan crystals dissolution. Optical density was measured with a microplate reader at a wavelength of 570 nm (Thermo Scientific Varioskan Flash, USA).

### Real-time quantitative PCR

TRIzol reagent was used to extract total RNA from A549 cells. Then, it was processed with cDNA synthesis by using a PrimeScript RT-PCR Kit (Takara). Real-time PCR was conducted on Applied Biosystems 7500 using the following thermocycling: 94 °C for 30 s, followed by 40 cycles of 94 °C for 5 s and 60 °C for 34 s. The mRNA levels of the target genes were normalized to the GAPDH mRNA level by using a 2^−△△CT^ method [31]. ΔΔCt = (Ct_target_ – Ct_GAPDH_) _treatment_ – (Ct_target_ – Ct_GAPDH_) _control_. Table 1 shows the primers and probes that we used.

**Table 1:** Primers and Probe Sequences for Real-time PCR

Gene	Primers and Probes	Sequence (5′ → 3′)
IL-6	Forward	CGGGAACGAAAGAGAAGCTCTA
	Reverse	CGCTTGTGGAGAAGGAGTTCA
	Probe	TCCCCTCCAGGAGCCCAGCT
TNF-α	Forward	AACATCCAACCTTCCCAAACG
	Reverse	GACCCTAAGCCCCCAATTCTC
	Probe	CCCCCTCCTTCAGACACCCTCAACC
COX-2	Forward	GAATCATTCACCAGGCAAATTG
	Reverse	TTTCTGTACTGCGGGTGGAAC
	Probe	TTCCTACCACCAGCAACCCTGCCA
GAPDH	Forward	GAAGGTGAAGGTCGGAGTC
	Reverse	GAAGATGGTGATGGGATTTC
	Probe	CAAGCTTCCCGTTCTCAGCC

### ELISA

H9N2 virus-infected A549 cells were treated with various concentrations of arctiin. After 24 h, culture supernatants were collected and centrifuged at 10,000 *g* for 15 min to remove the dead cells and cell debris. Next, the levels of proinflammatory cytokines in the culture supernatants were assayed using ELISA kits (IL-6 and TNF-α) in accordance with the manufacturers’ instructions (Multiscience, Hangzhou, China). The absorbance was measured at a wavelength of 450 nm with correction at 570 nm by a microplate reader (Multiskan FC, Thermo Fisher Scientific, MA, USA).

### Western blot analysis

Confluent monolayers of A549 cells in a six-well plate, infected with H9N2 viruses, were treated with various concentrations of arctiin. After 24 h, the cells were washed twice with PBS and lysed in ice-cold RIPA buffer (Beyotime, shanghai, China) supplemented with protease inhibitor cocktail (Sigma-Aldrich) and 1 mM phenylmethanesulfonyl fluoride (PMSF; Sigma-Aldrich). Cellular lysates were collected and centrifuged for 15 min at 10,000 *g* at 4 °C to remove debris. Then, protein concentration of the supernatant was measured by BCA Protein Assay Kit (Thermo Scientific, Waltham, MA, USA). Afterward, 20 μg of total protein from each sample was fractionated by 10% SDS-PAGE. After transfer of the resolved protein onto 0.2-μm PVDF membranes, the membranes were blocked with 5% nonfat milk and incubated with primary antibodies overnight at 4 °C. Then, the membranes were washed three times with 1 × TBST (Tris-buffered saline; 20 mM Tris-HCl PH7.4, 150 mM NaCl, 0.1% Tween 20) and further incubated with HRP-conjugated secondary antibody (Multiscience, Hangzhou, China) (1:5000 dilution) for 1 h at room temperature. The bands were detected by a chemiluminescence kit (PerkinElmer).

### Immunofluorescence staining

For Nrf2 nuclear translocation assay, A549 cells (1 × 10^4^ cells/well) seeded onto glass slides (9 mm, diameter) were incubated in 5% CO_2_ at 37 °C overnight. Then, A549 cells were infected with H9N2 viruses and treated with or without arctiin. After 24 h, the cells were washed three times with PBS, fixed with methanol for 20 min, and permeabilized with 0.5% Triton X-100 for 15 min. Then, they were blocked with 5% BSA for 30 min at room temperature and stained with rabbit polyclonal anti-Nrf2 antibody (1:200) overnight at 4 °C. Next day, the cells were incubated with FITC-conjugated goat anti-rabbit secondary antibody (1:200; MultiSciences, Hangzhou, China) for 1 h at room temperature, and cellular nuclei were stained with DAPI. The glass slides were sealed with glycerin, and imaging was performed with ZEISS Axio Imager.D2 microscope (ZEISS, Hamburg, Germany).

### Data analysis

One-way ANOVA followed by Tukey’s multiple comparison test was used for comparison between groups. Probability levels less than 0.05 (*P* < 0.05) were considered significant. SPSS 18.0 software (SPSS, Inc., Chicago, IL, USA) was used for data analysis, and variables were presented as mean ± standard deviation (SD).

## Results

### Structure, cytotoxicity and antiviral effects of arctiin

**Fig. 1:**
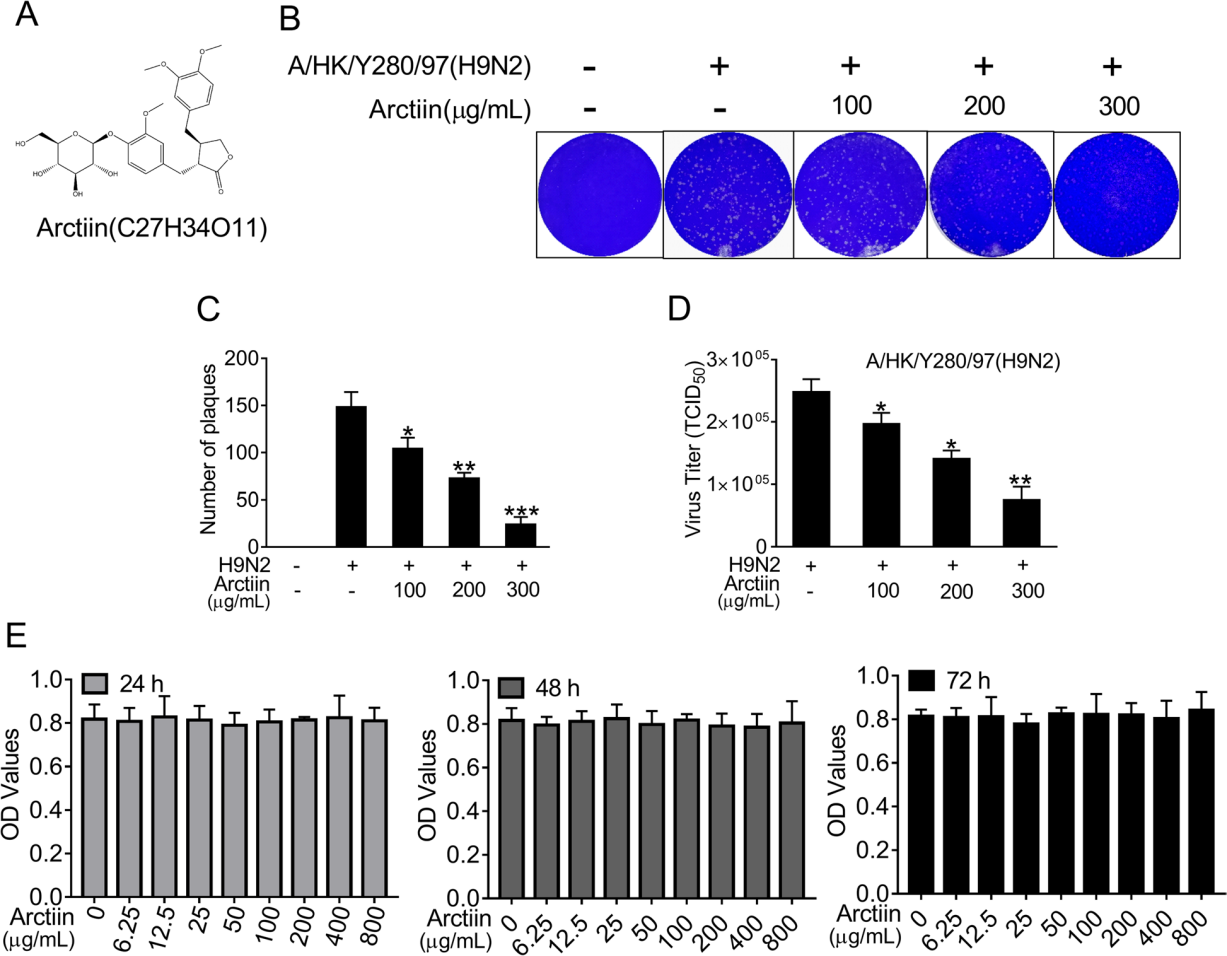
Cytotoxicity of arctiin on A549 cells. **A** Structure of arctiin. **B** Arctiin inhibited H9N2 virus-induced plaque formation in MDCK cells. **C** The number of plaques in (**B**) were showed. **D** Arctiin inhibited yield of H9N2 progeny viruses. A549 cells infected with H9N2 viruses (MOI = 0.1) were treated with or without arctiin (100–300 μg/mL). After 24 h, the culture supernatants were collected for progeny virus reduction assay. **E** Cytotoxicity of arctiin in A549 cells. A549 cells (5 × 10^4^ cells/mL) were treated with arctiin at various concentrations (0 to 800 μg/mL) for 24, 48 and 72 h. Then, MTT assay was carried out to investigate the cytotoxicity of arctiin. The results are expressed as means ± SD. ^*^p < 0.05; ^**^p < 0.01; ^***^p < 0.001 compared with H9N2 virus-infected alone

*Arctiin* isolated from *Arctium lappa L.* was confirmed by NMR spectroscopy. White powder, ESI-MS *m/z* 552.1 [M + NH_4_]^+^; ^1^H-NMR (CDCl_3_, 500 MHz), δ: 6.92 (1H, d, J = 8.0 Hz, H-5), 6.82 (1H, m, H-5′), 6.75 (1H, m, H-2), 6.64 (1H, m, H-2′), 6.56(1H, m, H-6), 6.49 (1H, m, H-6′), 4.80 (1H, s, Glu-1″), 4.12 (1H, m, H-9’a), 3.83 (1H, m, H-9’b), 3.79–3.70 (9H, m, −OMe), 2.86 (2H, m, H-7), 2.63 (1H, m, H-7’a), 2.55(1H, m, H-8), 2.47 (1H, m, H7’b); ^13^C-NMR (CDCl_3_, 125 MHz), δ: 178.6 (C-9), 150.0 (C-3), 149.2 (C-3′), 148.0 (C-4), 145.1 (C-4′), 133.6 (C-1), 130.4 (C-1′), 121.9 (C-6′), 121.0 (C-6), 118.0 (C-5), 113.4 (C-2′), 112.2 (C-2), 111.6 (C-5′), 102.4 (glu-1″), 76.0–77.3 (glu-2″, 3″, 4″, 5″), 71.2 (C-9′), 61.8 (C-6″), 56.0 (3, 4, 5′-OMe), 46.5 (C-8), 41.2 (C-8′), 38.1 (C-7′), 34.5 (C-7). The data were in accordance with the document of arctiin (Fig. 1A) [32]. Then, the antiviral effect against H9N2 influenza avian virus was confirmed by PRA and progeny virus reduction assay (Fig. 1B-D). Before the analysis of the pharmacological action, an MTT assay was employed to assess whether treatment with arctiin would induce direct cytotoxicity to A549 cells. Treatment with arctiin at a concentration ranging from 0 to 800 μg/mL for 24, 48 and 72 h did not affect the viability of A549 cells (Fig. 1E). And, we used doses of arctiin ≤300 μg/mL in the following experiments.

### Arctiin inhibits H9N2 avian influenza virus-mediated proinflammatory response

**Fig. 2:**
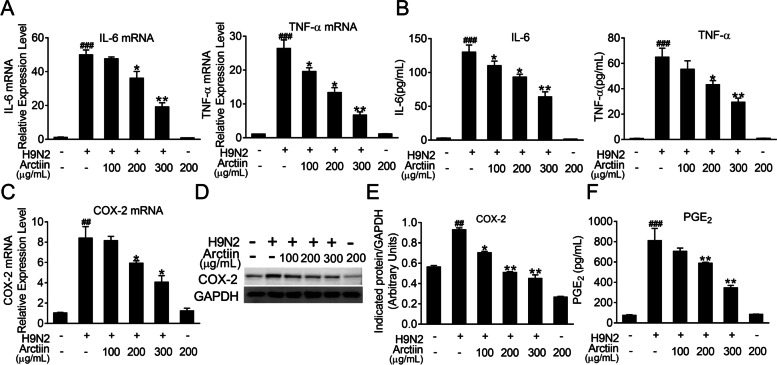
Effects of arctiin on H9N2 avian influenza virus (AIV)-induced inflammation. A549 cells infected with H9N2 AIVs (MOI = 0.1) were treated with or without arctiin (100–300 μg/mL) for 24 h. The cells were lysed by TRIzol to assess the mRNA levels of proinflammatory cytokines (**A**) and COX-2 (**C**). Protein levels of proinflammatory cytokines (**B**) in the culture supernatants and COX-2 expression (**D**) were measured by ELISA and western blot analysis, respectively. **E** Band intensities of COX-2 in (**D**) were normalized to GAPDH by densitometric analysis using ImageJ software. **F** The concentration of PGE_2_ in the culture supernatants was measured by ELISA. The results are expressed as means ± SD. ^##^*p* < 0.01; ^###^*p* < 0.001 compared with the control group; ^*^p < 0.05; ^**^p < 0.01 compared with H9N2 virus infection alone

Infection with H9N2 influenza avian viruses triggers intense inflammation, which results in disease severity [33]. Previous reports have indicated anti-inflammatory properties of arctiin in response to various stimuli [28, 34]. Therefore, we hypothesized that arctiin could reduce the expression levels of proinflammatory cytokines elicited by H9N2 viruses. As expected, the results showed that the increased mRNA levels of proinflammatory cytokines (including IL-6 and TNF-α) upon H9N2 virus infection were significantly suppressed by arctiin treatment (Fig. 2A). Consistently, elevated secretion of these proinflammatory mediators in the culture supernatant was also effectively inhibited by arctiin (Fig. 2B). It has been reported that COX-2 and its lipid metabolite PGE_2_ are critical inflammation mediators and associated with viral pneumonia progression [35]. Here, we found that treatment with arctiin decreased H9N2 virus-mediated expression of COX-2 at mRNA and protein levels in a dose-dependent manner (Fig. 2C-E). Meanwhile, production of PGE_2_ was detected by ELISA; as shown in Fig. 2F, treatment with arctiin significantly reduced H9N2-induced increased levels of PGE_2_. These results indicate that arctiin can attenuate H9N2 virus-mediated inflammation.

### Arctiin attenuates H9N2 avian influenza virus-mediated activation of P-JNK MAPK signaling

**Fig. 3:**
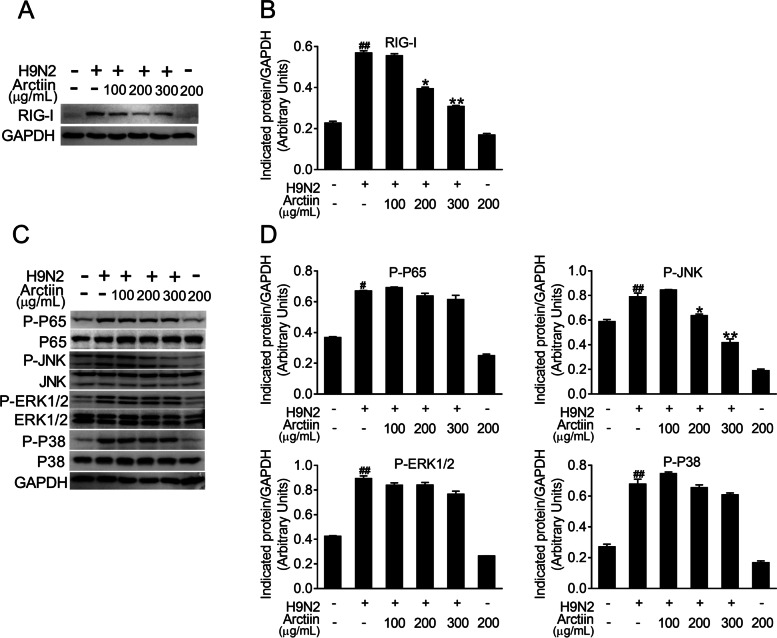
Effects of arctiin on H9N2 avian influenza virus (AIV)-activated host cellular signaling pathways. A549 cells infected with H9N2 AIVs (MOI = 0.1) were treated with or without arctiin (100–300 μg/mL) for 24 h. **A** Expression level of RIG-I was analyzed by western blot analysis. **B** Band intensities of RIG-I in (**A**) were normalized to GAPDH by densitometric analysis using ImageJ software. **C** Effects of arctiin on H9N2-mediated activation of NF-κB and MAPKs (ERK1/2, p38, and JNK) were detected by western blot analysis. **D** Band intensities of NF-κB and MAPKs in (**C**) were normalized to GAPDH by densitometric analysis using ImageJ software. The results are expressed as means ± SD. ^##^*p* < 0.01 compared with the control group; ^*^p < 0.05; ^**^p < 0.01 compared with H9N2 virus infection alone

H9N2 virus infection activates host cellular signaling cascades and thus triggers severe pneumonia [33]. To understand the anti-inflammatory mechanism of arctiin, we next focused on the effect of arctiin on H9N2 virus-mediated cellular signaling pathways. RIG-I is a PRR for recognition of influenza A virus-derived 5′ppp-RNA, resulting in downstream MAPK signaling pathway activation [9]. We therefore investigated the effects of arctiin on the expression of RIG-I during H9N2 virus infection. A549 cells infected by H9N2 virus markedly induced RIG-I expression. However, arctiin treatment of H9N2 virus-infected cells potently suppressed RIG-I expression (Fig. 3A and B). Considering the inhibitory effect of arctiin on RIG-I, it is possible that the downstream signaling pathways of RIG-I would be inhibited by arctiin treatment. Accordingly, western blot demonstrated that activation of JNK MAPK mediated by H9N2 viruses, but not NF-κB, ERK1/2, and p38 MAPK, was inhibited by arctiin (Fig. 3C and D). Therefore, these results indicate that arctiin dampens H9N2 virus-mediated inflammation by blocking the RIG-I/JNK signaling pathway.

### Arctiin treatment activates Nrf2 signaling in H9N2 avian influenza virus-infected cells

**Fig. 4:**
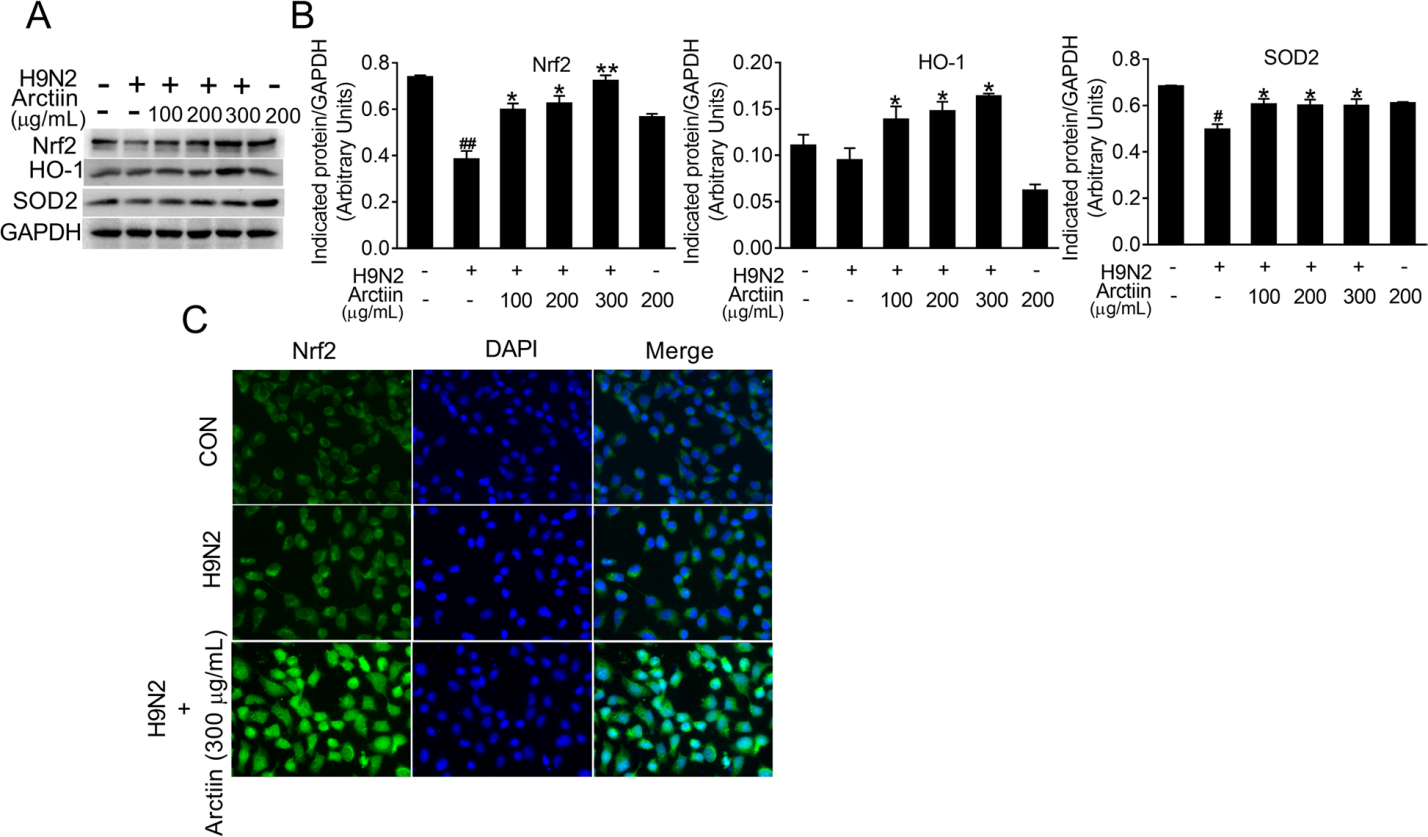
Effect of arctiin on Nrf2 signaling in H9N2 avian influenza virus (AIV)-infected A549 cells. **A** Western blot analysis of Nrf2 signaling (Nrf2, HO-1, and SOD2) in H9N2 virus-infected cells with or without arctiin (100–300 μg/mL) treatment for 24 h. **B** Band intensities of Nrf2, HO-1, and SOD2 in (**A**) were normalized to GAPDH by densitometric analysis using ImageJ software. **C** Effect of arctiin on Nrf2 nuclear translocation was investigated by immunofluorescence. A549 cells were inoculated with H9N2 AIVs (MOI = 0.1) for 2 h and then treated with arctiin (300 μg/mL). After 24 h treatment, the location of Nrf2 was detected by immunofluorescence. The results are expressed as means ± SD. ^#^*p* < 0.05; ^##^*p* < 0.01 compared with the control group; ^*^p < 0.05; ^**^p < 0.01 compared with H9N2 virus infection alone

Upon viral infection, the Nrf2 signaling pathway resolves excessive proinflammatory response [15]. To further clarify the anti-inflammatory role of arctiin in H9N2 virus infection, the effects of arctiin on Nrf2 signaling were investigated using western blot analysis. The results showed that Nrf2 expression significantly decreased in H9N2 virus-infected A549 cells. However, arctiin treatment reversed the inhibitory effect of H9N2 on Nrf2 expression (Fig. 4A and B). Moreover, the H9N2 virus-mediated the decreased in the main effectors of Nrf2 signaling (HO-1 and SOD2) was rescued by arctiin treatment (Fig. 4A and B). Moreover, immunofluorescence staining demonstrated that arctiin treatment increased the accumulation of nuclear Nrf2 in response to H9N2 virus infection (Fig. 4C). Therefore, we assume that the anti-inflammatory effects of arctiin may be associated with the activation of Nrf2 signaling.

### HO-1 inhibition reverses the inhibitory effects of arctiin on RIG-I/P-JNK MAPK signaling

**Fig. 5:**
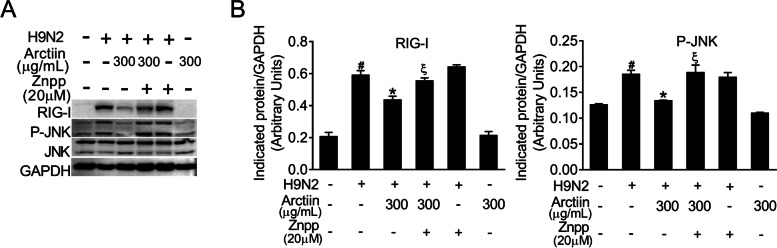
Abrogation of the inhibitory effects of arctiin on RIG-I/P-JNK MAPK signaling by HO-1 inhibition. **A** A549 cells were pretreated with Znpp (20 μM) for 2 h and then inoculated with H9N2 avian influenza virus at MOI of 0.1 for 2 h. The virus-infected cells were treated with arctiin (300 μg/mL) or in combination with Znpp (20 μM) treatment. After 24 h, the cells were lysed and western blot analysis of RIG-I and phosphorylated-JNK was done. **B** Band intensities of RIG-I and phosphorylated-JNK in (**A**) were normalized to GAPDH by densitometric analysis using ImageJ software. The results are expressed as means ± SD. ^#^*p* < 0.05 compared with the control group; ^*^p < 0.05 compared with H9N2 virus infection alone; ^ξ^*p* < 0.05 compared with H9N2 virus + arctiin (300 μg/mL) group

To further confirm whether the anti-inflammatory effect of arctiin is associated with the activation of Nrf2 signaling, we performed western blotting to detect the RIG-I/JNK signaling in H9N2 virus-infected A549 cells with Znpp (HO-1 inhibitor) or in combination with arctiin treatment. Notably, the reduction of H9N2 virus-mediated RIG-I expression by arctiin was abrogated in cells with Znpp pretreatment (Fig. 5A and B). Meanwhile, the inhibitory effect of arctiin on the activation of JNK MAPK was also reversed by HO-1 inhibition (Fig. 5A and B). Therefore, arctiin inhibits H9N2 virus-mediated RIG-I/P-JNK MAPK signaling via activation of Nrf2 signaling.

### HO-1 inhibition abrogates the anti-inflammatory effects of arctiin in H9N2 avian influenza virus-infected A549 cells

**Fig. 6:**
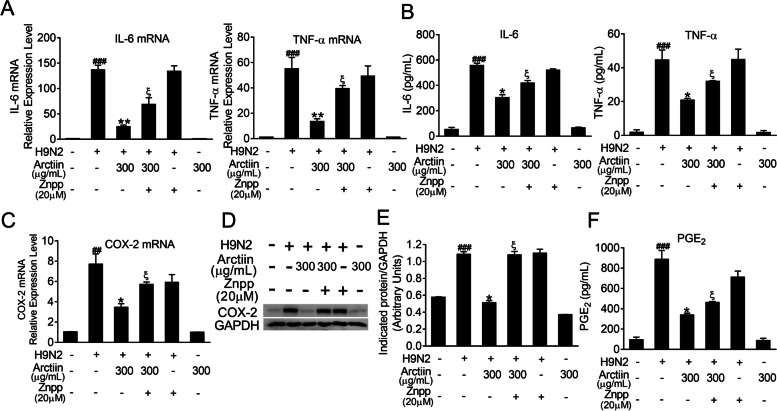
Reversing the anti-inflammatory effects of arctiin in H9N2-avian influenza virus (AIV)-infected A549 cells by HO-1 inhibition. A549 cells were pretreated with Znpp (20 μM) for 2 h followed by H9N2 AIVs (MOI = 0.1) infection. Two hours after inoculation, the cells were treated with arctiin (300 μg/mL) or in combination with Znpp (20 μM) treatment for 24 h. **A-B** mRNA (**A**) and protein (**B**) levels of proinflammatory cytokines were analyzed by qPCR and ELISA, respectively. **C-D** mRNA (**C**) and protein (**D**) levels of COX-2 were analyzed by qPCR and western blot analysis, respectively. **E** Band intensities of COX-2 in (**D**) were normalized to GAPDH by densitometric analysis using ImageJ software. **F** The concentration of PGE_2_ in the culture supernatants was measured by ELISA. The results are expressed as means ± SD. ^##^*p* < 0.01; ^###^*p* < 0.001 compared with the control group; ^*^p < 0.05; ^**^p < 0.01 compared with H9N2 virus infection alone; ^ξ^*p* < 0.05 compared with H9N2 virus + arctiin (300 μg/mL) group

Given that the inhibitory effect of arctiin on RIG-I/JNK MAPK signaling was abrogated by HO-1 inhibition, we next investigated whether the anti-inflammatory effect of arctiin on H9N2 virus-mediated expression of proinflammatory mediators was reversed in cells with Znpp pretreatment. As shown in Fig. 6A and B, Znpp pretreatment in H9N2 virus-infected cells abrogated the inhibitory effect of arctiin on the mRNA levels and protein levels of proinflammatory cytokines, including IL-6 and TNF-α. Similarly, the reduction of COX-2 and PEG_2_ expression by arctiin was reversed by Znpp pretreatment (Fig. 6C-F). Therefore, these results suggest that the anti-inflammatory effects of arctiin involve Nrf2/HO-1 activation, which results in suppression of H9N2 virus-induced RIG-I/JNK MAPK signaling.

## Discussion

Globally, the circulation of H9N2 AIVs among avian species provided gene segments to generate novel lethal viruses (e.g., H5N1 and H7N9), posing a serious threat to public health [5, 6]. Rapid mutation and reassortment of viruses have led to vaccine inefficacy and resistance to antiviral agents (adamantane and oseltamivir) [36], so there are few effective therapy options for influenza diseases. Arctiin is the effective component of *Arctium lappa L.* that has been reported to possess anti-inflammatory, antiviral, antioxidant, and antitumor properties [28, 36–39]. However, the effect of arctiin on H9N2 AIV infection has not yet been reported, and the corresponding mechanism needs to be clarified. Here we reported that arctiin reduced H9N2 AIV replication. Our further experiments revealed that arctiin treatment reduced H9N2 virus-mediated inflammation via activation of the Nrf2/HO-1 pathway, resulting in the blockade of RIG-I/P-JNK signaling. According to our study, the activation of the Nrf2/HO-1 pathway is likely to contribute to the antiviral and anti-inflammatory effects of arctiin; hence, arctiin may provide an alternative strategy to limit transmission of H9N2 viruses.

Our results showed that arctiin inhibited H9N2 virus replication, as evidenced by the results of PRA and progeny virus reduction assay. A previous study showed that arctiin treatment could increase virus-specific antibodies [29], but the mechanism involved in its effects on host signaling linked to antiviral effects was not reported. The replication of IAV, including H9N2 virus, is dependent on the activation of host cellular signaling [40]. During viral infection, the activation of NF-κB and ERK1/2 MAPK signaling has been reported to support viral ribonucleoprotein nuclear export [11, 40]. Besides, JNK signaling is also involved in IV life cycle. Inhibition of JNK by its specific inhibitor SP600125 decreased synthesis of vial genomic RNA (vRNA) and structural proteins [41]. Viral nucleoprotein hijacked cellular Filamin A, resulting in activation of the JNK pathway to promote virus efficient replication. In vivo studies demonstrated that JNK inhibition reduced virus titer and alleviated virus-mediated lung injury [42]. Our present study showed that arctiin suppressed H9N2 virus-mediated JNK activation. Therefore, we supposed that the mechanism by which arctiin exerted antiviral effects on H9N2 virus might be related to its inhibition of the JNK pathway.

The transcription factor Nrf2 is a restriction factor to limit the replication of multiple viruses, including IAV. The anti-influenza mechanism of Nrf2 involves the inhibition of virus entry [43]. Moreover, the antioxidative enzyme HO-1, a downstream effector of Nrf2, has been reported to strengthen generation of type I IFNs and IFN-stimulated genes (ISGs) (IFIT1, OAS1, and IFITM3), leading to reduced IAV titers [20, 44]. Our findings demonstrated that the impaired expression of Nrf2 and Nrf2 target genes (HO-1 and SOD2) by H9N2 virus was reversed by arctiin treatment. As indicated above, the anti-H9N2 virus effects of arctiin may be associated with the activation of the Nrf2 signaling pathway.

In addition to its anti-H9N2 virus effects, arctiin suppressed H9N2 virus-induced inflammation. The anti-inflammatory properties of arctiin have been reported previously. Arctiin treatment dose-dependently reduced LPS-mediated upregulation of IL-6, TNF-α, and IL-1β, as well as reduction of PGE_2_ and NO [45]. Besides, arctiin showed the potential therapeutic effects in depression by attenuation of neuroinflammation [34]. However, this is the first report to investigate the regulatory role of arctiin in H9N2 virus-mediated inflammation. Here, we found that arctiin treatment reversed H9N2 virus-elicited upregulation of proinflammatory mediators (IL-6, TNF-α, COX-2, and PGE_2_). Accumulated evidence has demonstrated that the magnitude and duration of inflammation are linked to the outcome of influenza diseases [46]. An appropriate immune response is capable of controlling viral infection without simultaneously leading to lung damage [46]. Highly pathogenic AIVs (H5N1 and H7N9)-triggered viral pneumonia with robust cytokine production resulted in rapid progression of acute lung injury with fatal termination [47, 48]. The development of acute respiratory distress syndrome in mice challenged by H9N2 viruses was related to local and systemic hyperactive inflammation [49]. Moreover, reduction of inflammation effectively ameliorated H9N2 virus-mediated lung pathology and lung injury [33].

Initiated the expression of cytokines is firstly dependent on virus recognition by cellular PRRs, which results in the activation of multiple signaling pathways [7]. In response to viral infection, the cytosolic sensor RIG-I has been reported to be responsible for JNK activation [50]. JNK signaling cascade transduction mediates phosphorylation of its downstream transcription factor c-jun, which is involved in numerous biological processes, such as proliferation, survival, and inflammation [51, 52]. IV-induced inflammation has been linked to the activity of the JNK/c-jun pathway [53]. IV-mediated upregulation of proinflammatory cytokines (IL-6, TNF-α, and CCL5) is JNK-dependent [53]. Moreover, JNK signaling also participates in IV-triggered production of lipid mediators (COX-2 and PGE_2_) [54]. Blockade of JNK signaling significantly ameliorated influenza-associated pneumonia and thus improved high mortality elicited by lethal influenza infections [51]. Pharmacological inhibition of c-jun has been shown to effectively reduce H5N1 virus-induced pneumonia [51]. Our results showed that arctiin treatment decreased the IV-mediated activation of JNK signaling. Based on the evidence shown above, we suppose that the inhibitory effects of arctiin on IV-induced inflammation are based on the suppression of JNK activation. Interestingly, we found that the inhibitory effect of arctiin on H9N2 virus-induced activation of JNK was reversed by an HO-1 inhibitor, indicating that the anti-inflammatory properties of arctiin depend on the activation of Nrf2/HO-1 signaling. Indeed, previous reports have suggested that the activation of Nrf2/HO-1 signaling has capacity to inhibit NF-κB, P38, and JNK signaling pathways and thus prevent IV-mediated uncontrolled proinflammatory reaction [55–57]. Nrf2 or HO-1 inhibition abrogated the anti-inflammatory effects exerted by various bioactive agents [58, 59]. A recent study has revealed that arctiin prevented triptolide-induced acute live damage through activation of Nrf2 signaling [60]. Likewise, our findings showed that the blockade of HO-1 reversed the suppression effect of arctiin on H9N2 virus-elevated proinflammatory mediators (IL-6, TNF-α, COX-2, and PGE_2_). Therefore, we concluded that the activated Nrf2/HO-1 pathway is likely to contribute to the anti-inflammatory properties of arctiin in response to H9N2 virus infection.

## Conclusions

In conclusion, we showed that the lignan glycoside arctiin from *Arctium lappa L.* activated Nrf2/HO-1 signaling, leading to inhibitory effects on RIG-I/JNK signaling. It showed potential inhibitory effects on replication of H9N2 virus and H9N2 virus-mediated inflammation, so it may be a novel candidate for the development of anti-influenza agents.

## Supplementary Information

**Additional file 1.**

## Data Availability

The datasets used and analyzed during the current study are available from the corresponding author on reasonable request.

## Abbreviations

AIV: Avian influenza virus
ATCC: American Type Culture Collection
HO-1: Heme oxygenase-1
IFN-β: Interferon beta
IL-6: Interleukin 6
IL-8: Interleukin 8
IP-10: Interferon-gamma induced protein 10
IV: Influenza virus
MOI: Multiplicities of infection
Nrf2: NF-E2-related factor 2
PRA: Plaque reduction assay
PRR: Pattern recognition receptor
SD: Standard deviation
SOD2: Superoxide dismutase 2
TNF-α: Tumor necrosis factor alpha
