# Supplementary figures and images for: Arctiin suppresses H9N2 avian influenza virus-mediated inflammation via activation of Nrf2/HO-1 signaling
Source: BMC Complement Med Ther. 2021 Nov 26;21:289. doi: 10.1186/s12906-021-03462-4 (PMC8620712; doi:10.1186/s12906-021-03462-4)

Figure 2D


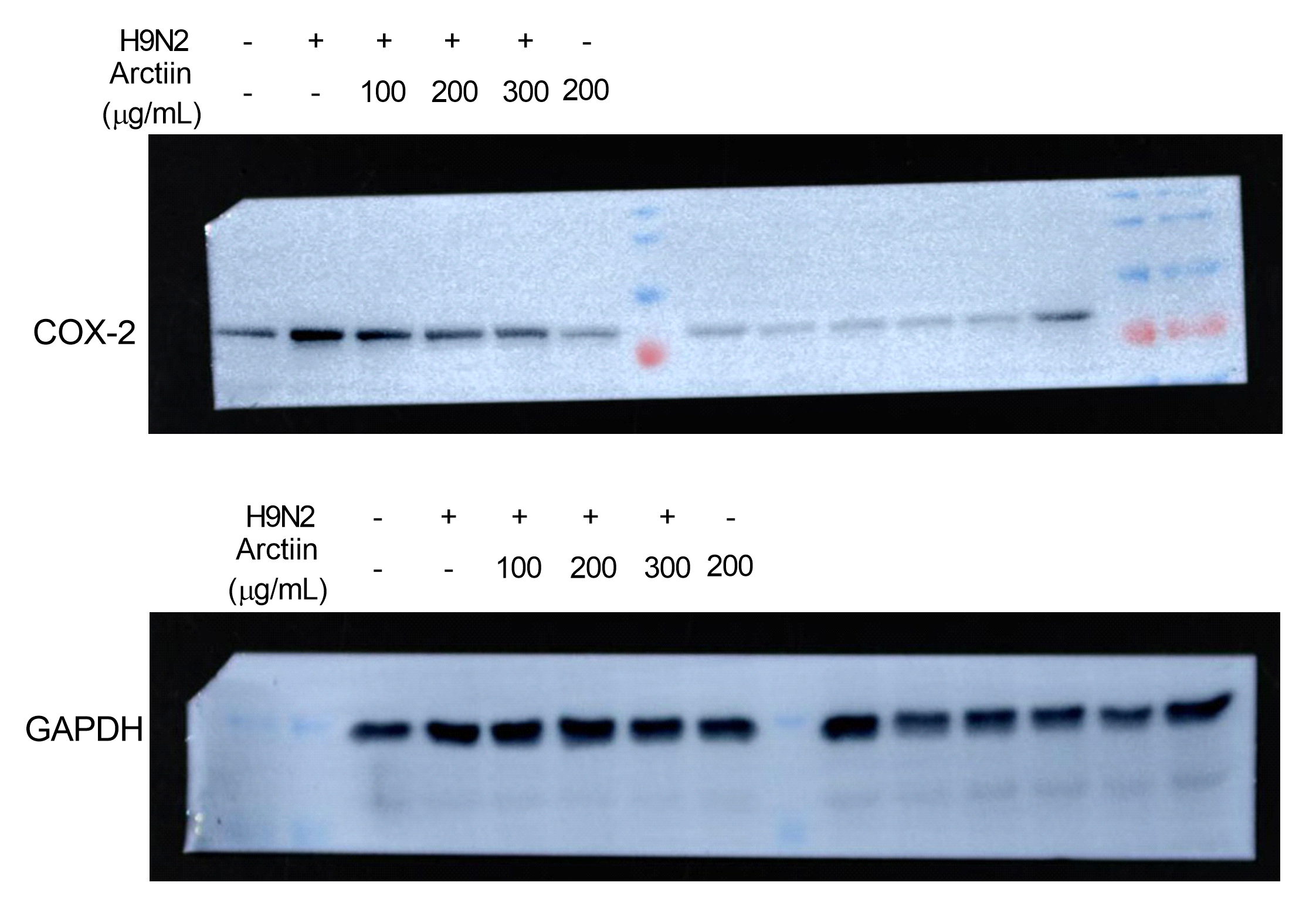


Figure 3A


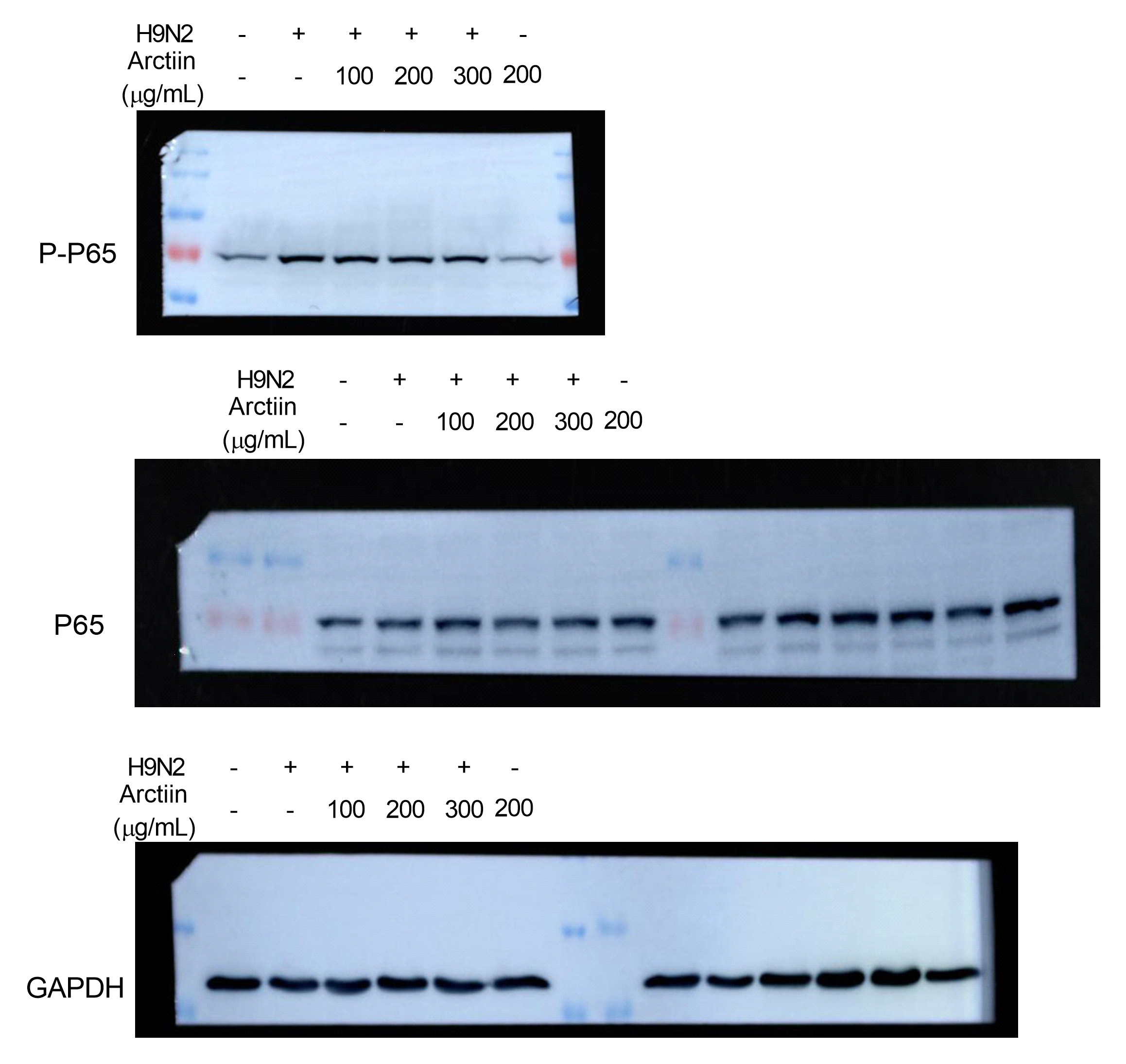


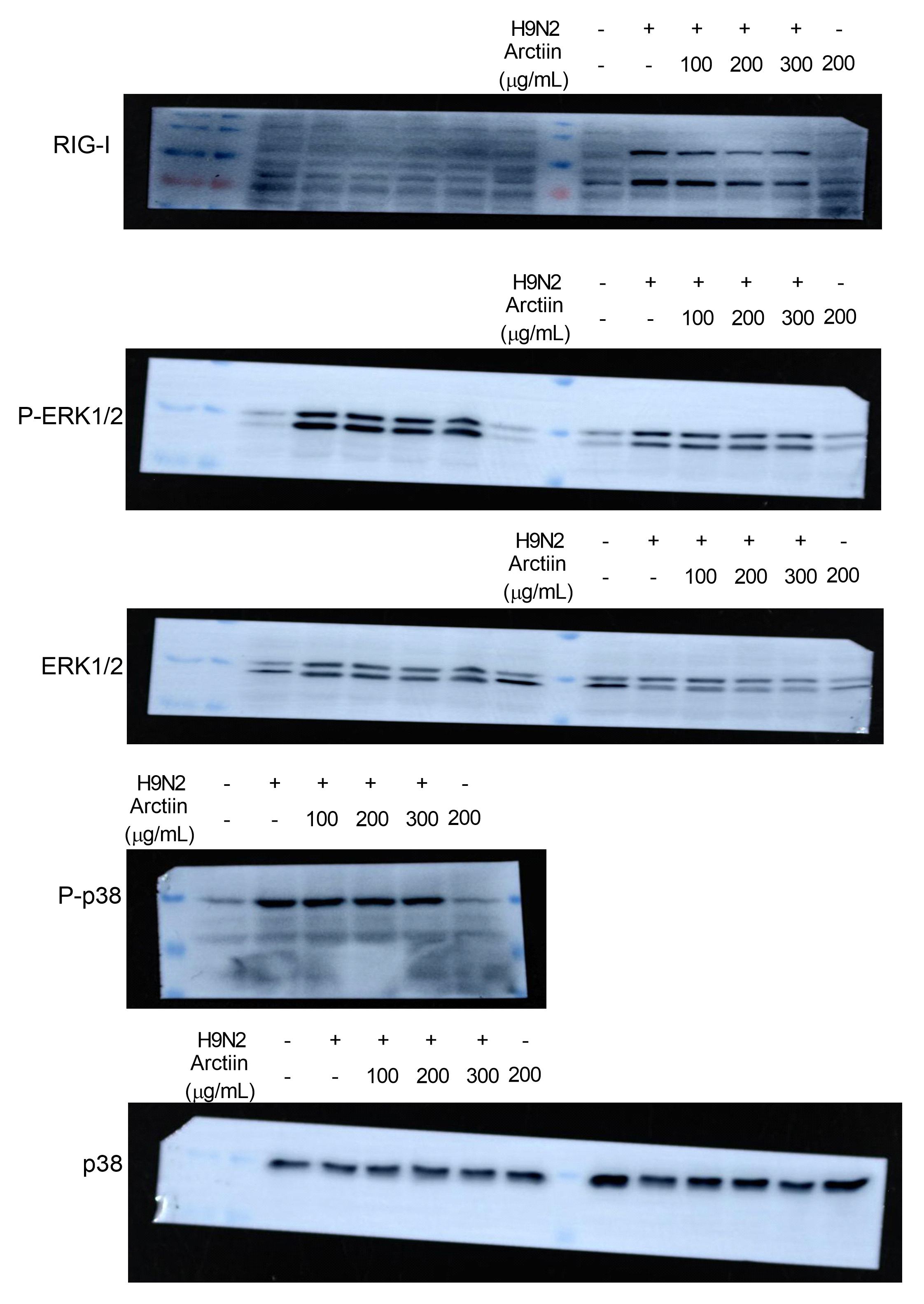
Figure 3C

Figure 3C


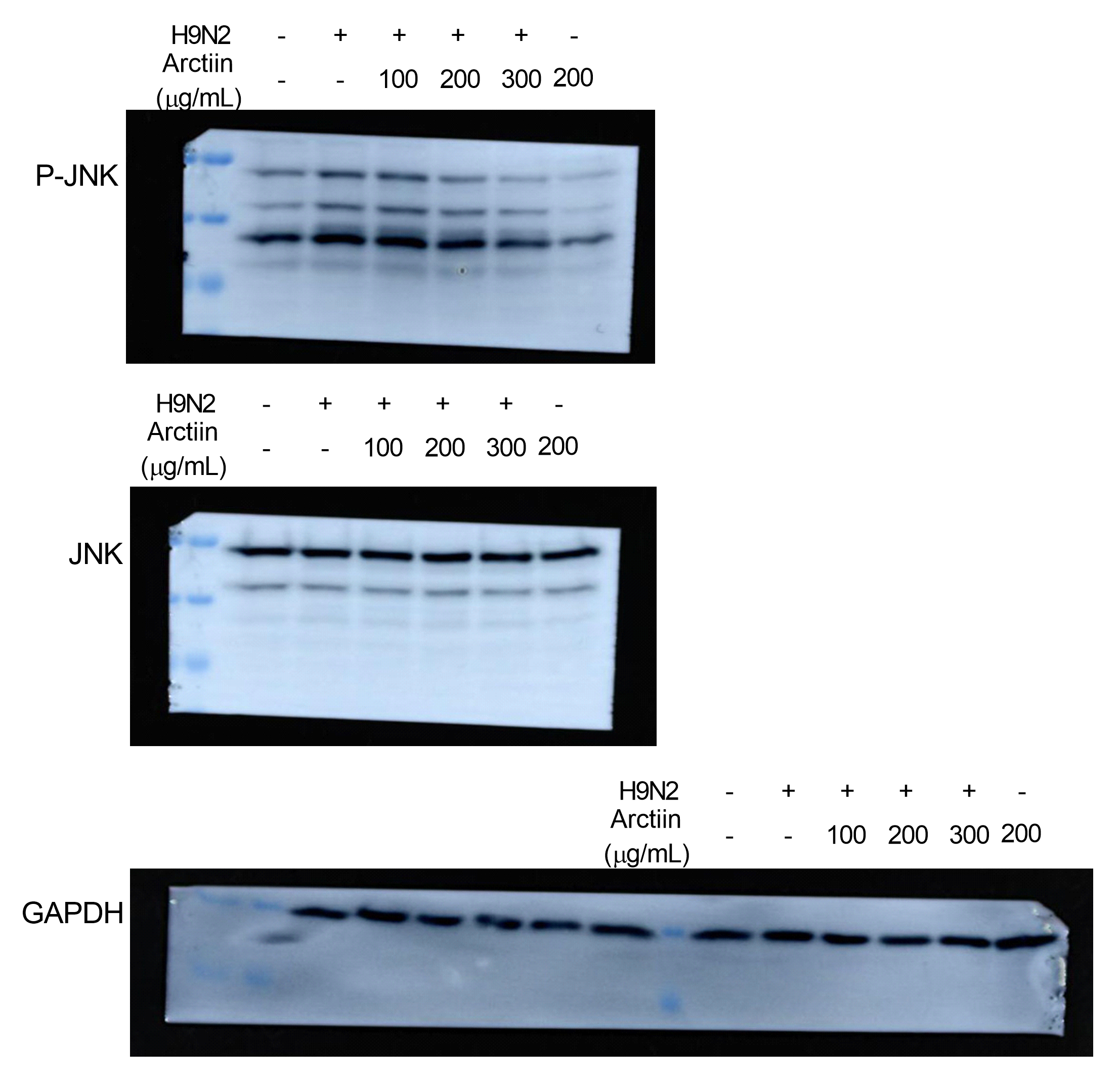


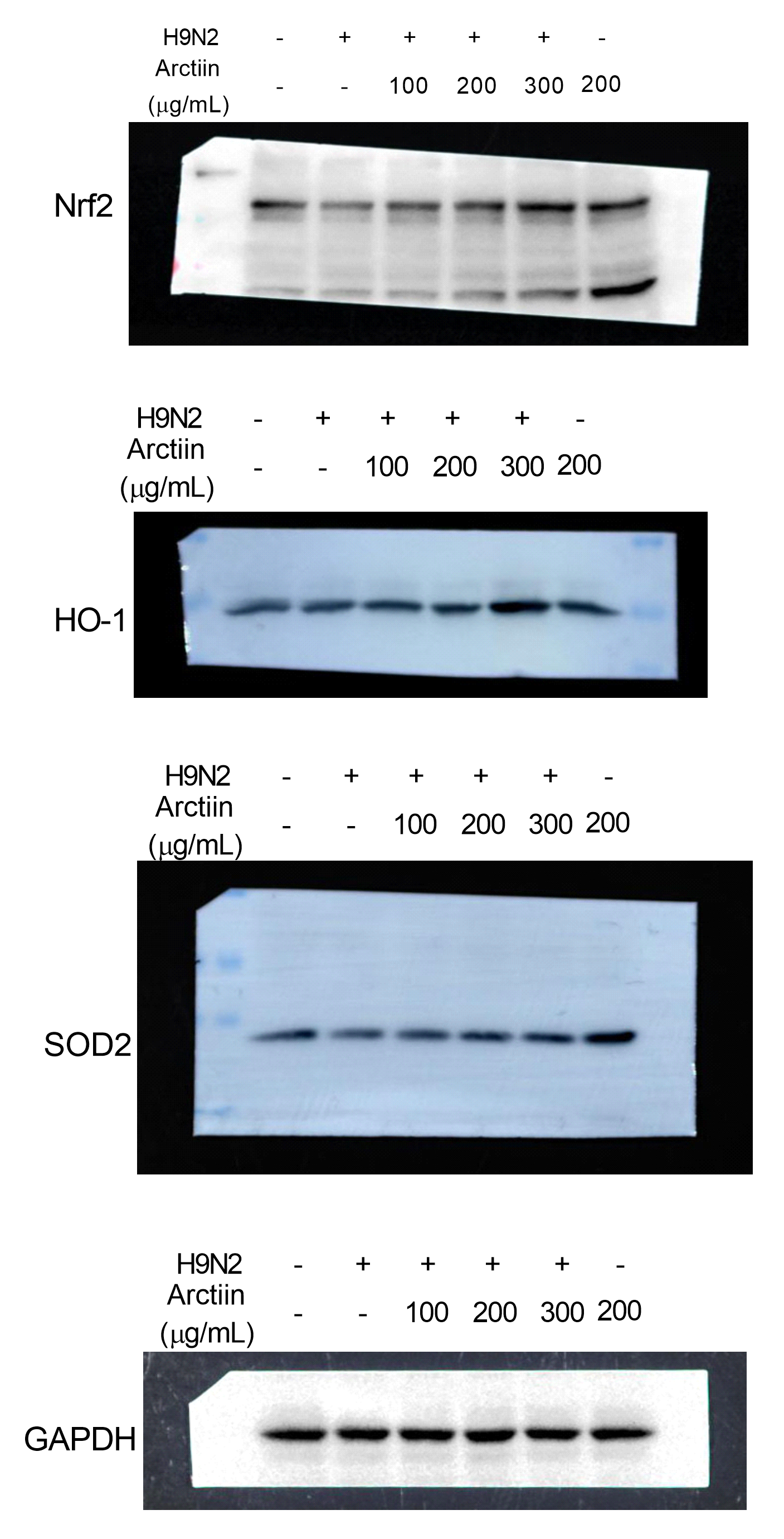
Figure 4A

Figure 5A


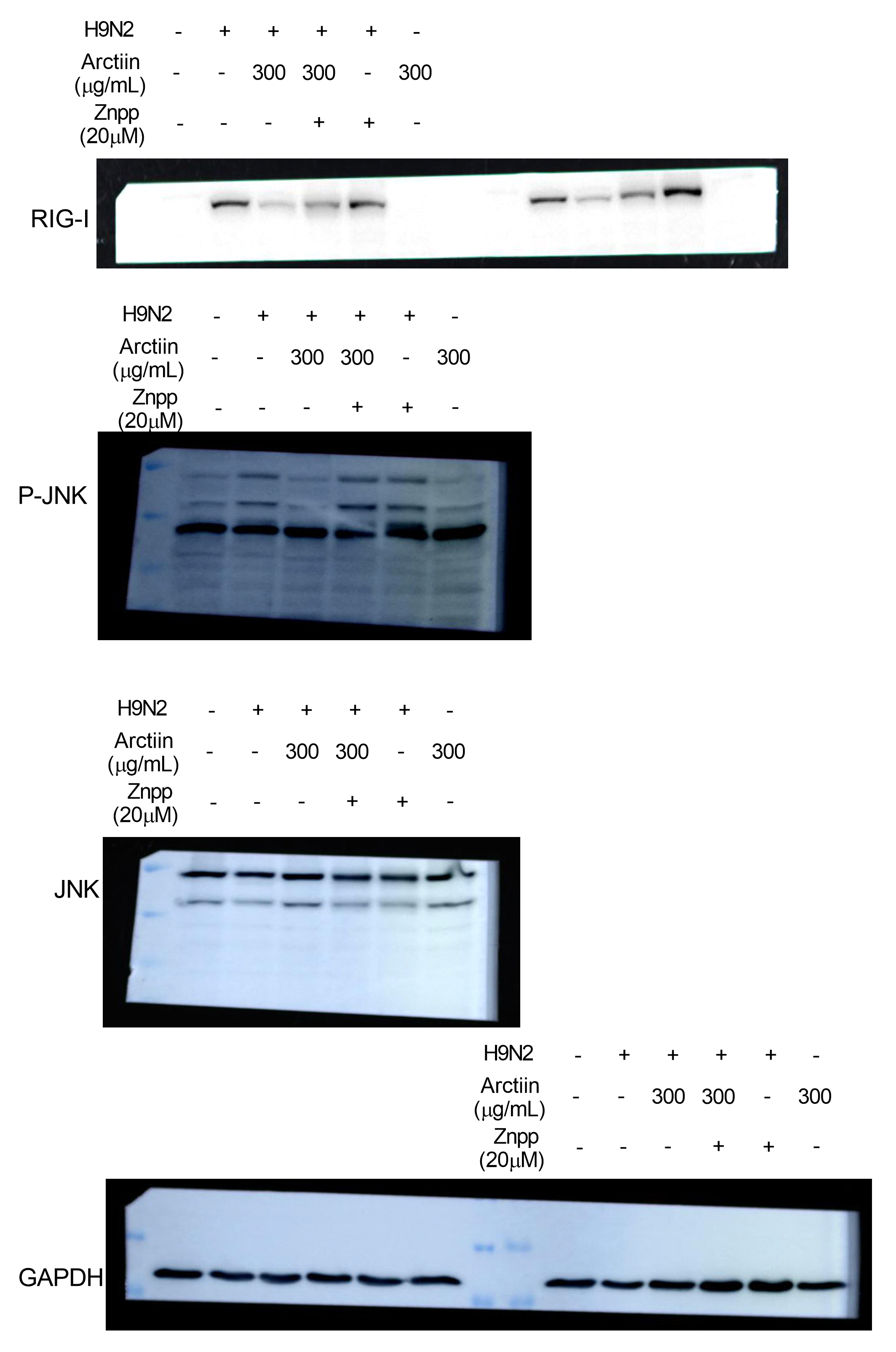


Figure 6D


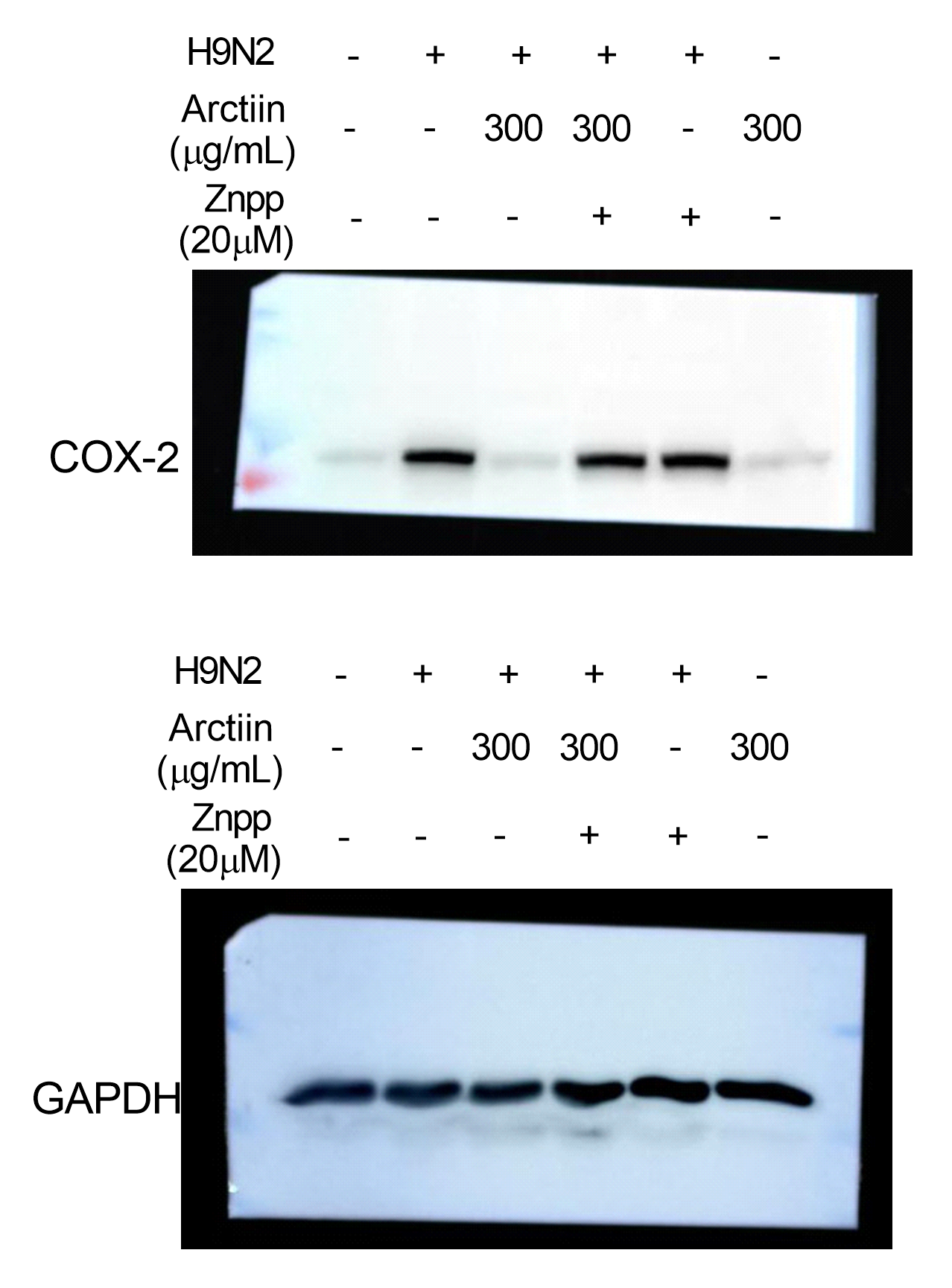

Supplement: Supplementary file 1 — Additional file 1. [file 12906_2021_3462_MOESM1_ESM.doc]
